# Can environmental conditions experienced in early life influence future generations?

**DOI:** 10.1098/rspb.2014.0311

**Published:** 2014-06-22

**Authors:** Tim Burton, Neil B. Metcalfe

**Affiliations:** Institute of Biodiversity, Animal Health and Comparative Medicine, College of Medical, Veterinary and Life Sciences, Graham Kerr Building, University of Glasgow, Glasgow G12 8QQ, UK

**Keywords:** parental effects, developmental plasticity, epigenetics, carry-over effects, germline, trans-generational effects

## Abstract

The consequences of early developmental conditions for performance in later life are now subjected to convergent interest from many different biological sub-disciplines. However, striking data, largely from the biomedical literature, show that environmental effects experienced even before conception can be transmissible to subsequent generations. Here, we review the growing evidence from natural systems for these cross-generational effects of early life conditions, showing that they can be generated by diverse environmental stressors, affect offspring in many ways and can be transmitted directly or indirectly by both parental lines for several generations. In doing so, we emphasize why early life might be so sensitive to the transmission of environmentally induced effects across generations. We also summarize recent theoretical advancements within the field of developmental plasticity, and discuss how parents might assemble different ‘internal’ and ‘external’ cues, even from the earliest stages of life, to instruct their investment decisions in offspring. In doing so, we provide a preliminary framework within the context of adaptive plasticity for understanding inter-generational phenomena that arise from early life conditions.

## 1. Introduction

Environmental factors, experienced even during the very earliest stages of life, have the potential to cause irreversible developmental changes. Consequently, an individual can ‘acquire’ any number of phenotypes, often with long-term consequences for performance [1]. For example, recent studies in wild vertebrates have revealed that conditions experienced in early life can have dramatic consequences for reproductive success years or even decades later [2–4]. However, striking evidence, much of which is found within the biomedical and epidemiological literature and which may not be readily apparent to ecologists, shows that the repercussions of conditions experienced during early development may not be limited to the individuals who experience them first-hand, but may affect the generations to follow [5–7]. Maternal or paternal (hereafter ‘parental’) effects on offspring have been the subject of immense interest within the fields of ecology and evolution [8,9]. However, often implicit within this field is the assumption that any environmental influence on such effects is driven by the environment experienced by the parental generation when adult, at the time of reproduction. Here, we explicitly focus instead on parental effects that can be linked to their environment in ‘early life’, defined hereafter as the period from before conception to the end of juvenile growth and the start of sexual maturation. By drawing from the biomedical literature and using supportive examples from natural systems where available, we review the diverse causes and consequences of trans-generational effects that can be linked to this early life period of the parental generation, focusing on why early life might be so sensitive to environmental perturbation. We also discuss the findings of several recent theoretical models of developmental plasticity that are relevant to this subject, thereby outlining a preliminary framework for understanding how parents might use cues from the external environment and also from the development of their own somatic state in making investment decisions in offspring that can have their origin in the very early stages of life. We conclude with suggestions for future work that will enable a more thorough examination of these phenomena for biologists.

## 2. Defining inter-generational transfer

**Figure 1.**
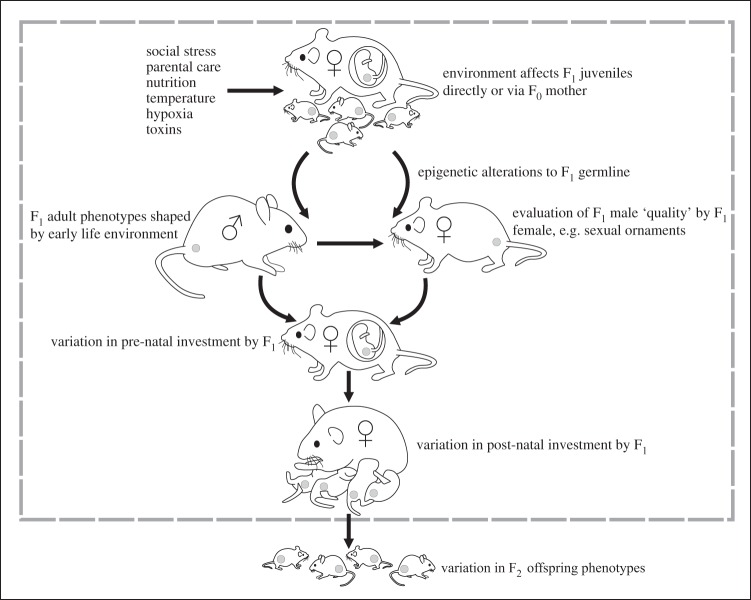
Pathways through which early life experiences of parents can affect offspring development. Environmental variation affects the parental generation, either directly on F_1_ juveniles or indirectly when they are gametes/fetuses within the F_0_ mother, leading to epigenetic alterations in the F_1_ germ cells (grey circle) which are then transmitted to offspring (F_2_) and induce phenotypic variation. Alternatively, or likely in combination with these direct epigenetic effects, early life experiences of F_1_ parents induce long-term phenotypic changes that affect their pre-and-post-natal investment in F_2_ offspring. Such effects may also result in changes in the ‘quality’ of F_1_ fathers as assessed by F_1_ females at the time of mating, leading to differential pre- and/or post-natal investment by F_1_ mothers. Effects confined to the grey box are not considered to be inter-generational effects as defined in the text. Adapted from [10].

The terminology used to describe the transmission of parental effects that stem from early life conditions can be varied, reflecting whether or not individuals in later generations are exposed directly to the environmental factor in question (e.g. [6]). Here, we adopt a simplified approach and refer to inter-generational, trans-generational and multi-generational effects interchangeably. However, it is important to clarify which is the exposed generation, especially when effects are seen in grand-offspring. If early post-natal conditions affect an individual (here termed the F_1_ generation, for reasons that will become apparent), with effects that are subsequently seen in its offspring (the F_2_ generation), it indicates an inter-generational effect due to early life conditions experienced by the F_1_ parent (figure 1). However, if these early life conditions are shaped by the preceding (F_0_) generation (e.g. through their choice of breeding location or intensity of parental care), then the variation in early life environment experienced by the F_1_ may be partially generated by variation in the environment experienced earlier in life by their F_0_ parents, pushing the root cause of the inter-generational effect back a generation. A further complication in terminology is caused by pre-natal effects. For example, if a pregnant female (F_0_) experiences an environmental perturbation which elicits a phenotypic response in her developing young (the F_1_ generation), we do not consider this as an inter-generational effect, as the embryo or fetus could be said to have experienced the change in environment directly (e.g. through a change in nutritional provisioning in the womb). It would only become an inter-generational effect if it resulted in a change in the F_2_ generation (figure 1).

## 3. The transmission of early life environmental effects across generations: evidence from human, animal and plant studies

A major reason underlying the recent interest in the generation-spanning effects of early life environmental conditions was the recognition among epidemiologists that the apparent heritability of human cardio-vascular and metabolic diseases might in fact stem from ‘programming’ phenomena initiated by stressors experienced early in the life of recent ancestors [5,6]. For example, several longitudinal analyses of human populations revealed that conditions during an F_0_ mother's pregnancy could alter the birth characteristics and/or later-life health of her F_2_ grandchildren [11–13]. However, such effects are not necessarily restricted to the maternal lineage nor first generation offspring: decreased lifespan has been reported in men whose paternal grandfather experienced poor nutrition during childhood [14]. Experimental data from laboratory model rodents, such as rats and mice, have corroborated these findings: traits linked with cardio-vascular, metabolic and neurological diseases may be ‘programmed’ by early life experiences of the parent and transmitted, by both parental lineages, to subsequent generations [10,15–17]. Preliminary evidence is now emerging for similar effects in animals and plants from natural populations that stem from numerous causative agents, affect a wide range of offspring traits, appear to be important for offspring reproductive success and can affect entire cohorts with lasting consequences for population-level processes (table 1). For example, in a well-controlled experimental study on the beetle *Tribolium castaneum*, experimental populations were initiated from larvae that had been reared on either high- or low-quality food; these were then allocated after metamorphosis to high or low food ‘colonizing’ environments, in which they (and their descendants) remained. Two to three full generations later, rates of cannibalism (a strategy to deal with low food) were the highest (and densities lowest) in populations derived from individuals that had originally developed in poor food habitats, irrespective of the food environment experienced thereafter [22].

**Table 1.** Experimental examples of environmental factors that can generate inter-generational effects by influencing parental development in early life. Also shown are the phenotypic responses in offspring and the number of generations over which an effect was demonstrated. We searched for studies that explicitly manipulated the early life environment (i.e. from the gamete stage until the point when individuals began the maturation process) of the parental generation and then measured offspring phenotypes for one or more generations. Correlative epidemiological studies are excluded.

environmental manipulation during parental development (F_0_)	offspring generations affected	effect on offspring	species	references
plants				
salt and heat stress	F_1_	time of flowering, salt tolerance	*Arabidopsis*	[18]
heavy metal exposure	F_2_	heavy metal tolerance	rice	[19]
arthropods				
temperature	F_1_	size	butterfly	[20]
nutrition level	F_1_	size	soil mite	[21]
dietary composition	F_2_–F_3_	foraging strategy, population growth rate and carrying capacity	flour beetle	[22]
nutrition level	F_1_	growth, development rate, immunity	butterfly	[23]
dietary composition	F_1_	size, development rate	fruit fly	[24]
dietary composition	F_1_	development rate, reproductive output, nutrient metabolism	fruit fly	[25]
hypoxia	F_1_	size, metabolic rate	water flea	[26]
fishes				
nutrition level	F_1_	size, growth	cichlid	[27]
birds				
nutrition level	F_1_	size	zebra finch	[28]
nutrition level	F_1_	reproductive success	zebra finch	[29]
nutrition level	F_1_	body condition	zebra finch	[30]
photoperiod	F_1_	growth, competitive ability, learning ability	chicken	[31]
social isolation	F_1_	stress response, growth, learning ability	chicken	[32]
disturbance	F_1_	personality type	quail	[33]
mammals				
nutrition level	F_1_	birth weight	vole	[34]
olfactory behavioural conditioning	F_2_	neuroanatomical alterations, sensitivity to olfactory cues	mouse	[35]
social environment	F_1_	alloparental interaction	prairie vole	[36]
nutrition level	F_1_	birth weight, growth	hamster	[37]
nutrition level	F_1_ and F_2_	F_1_ growth, F_2_ birth weight, survival	hamster	[38]

## 4. Mechanisms underlying the transmission of early environmental effects across generations

The inheritance of epigenetic alterations to gene expression is gaining popularity in biomedicine as a mechanistic explanation for the transmission of early environmental effects from parents to offspring [7]. During development, different cells and tissues acquire different profiles of gene expression, and it is thought that this is partially a consequence of environmentally induced changes to the genome (e.g. via methylation of DNA, histone modification or the production of small non-coding RNA molecules) [39,40]. For most cell types, these epigenetic ‘marks’ become fixed once cells differentiate or exit the cell cycle [39,40], enabling the production from the same genotype of different cellular phenotypes that are maintained throughout life (for recent reviews, see [10,41,42]). In mammals, environmentally induced alterations to the epigenome had been thought to be single generation entities because ‘reprogramming’ events during gamete production, and again shortly after fertilization, mean that embryonic development should begin with an epigenetically ‘blank canvas’ [39,40]. However, these reprogramming events are now thought to be incomplete, since diet, stress and other environmental factors experienced in early life—even prior to fertilization—can induce changes in DNA methylation/gene expression that (in the absence of the initial stimulus) are also observed in subsequent generations (figure 1) [15,31,32,35,43–45].

Several lines of evidence suggest that the ‘early life’ period, from pre-conception and extending through development, is particularly sensitive to the induction and cross-generational transmission of environmental effects on the epigenome. Firstly, epigenetic alterations that arise around the time of conception or during early embryogenesis can potentially affect a high proportion of cells (including germline cells, the embryonic presursors of gametes) in the fully grown organism. By contrast, when epigenetic alterations occur in fully differentiated adult cells they remain restricted to those cells. Secondly, the epigenomes of early embryonic cells seem particularly sensitive to environmental influences because they show relatively high expression levels of the regulatory ‘machinery’ involved in epigenetic alterations (e.g. molecular regulators of DNA methylation, [41]), and altered DNA methylation patterns that are acquired during development can seemingly be maintained throughout life: retrospective studies on human adults have linked the methylation of genes important for growth, metabolism and the response to stress with environmental conditions experienced by those individuals during gestation or childhood [46–49]. Thirdly, the majority of studies in model rodent systems in which inter-generational effects appear to be mediated via early life effects involve an environmental perturbation acting on a pregnant female (F_0_) that is coincident with the epigenetic reprogramming events that are occurring in the developing germ cells of her fetus (F_1_) [17]. For example, the inter-generational effects of nutrient restriction of F_0_ rats were negligible if the restriction occurred during the first half of pregnancy, whereas if nutrition was restricted in the second half of pregnancy the F_2_ were smaller at birth, had higher basal levels of cortisol and were less sensitive to stress [50]. The susceptibility of early development to the inter-generational transmission of epigenetic alterations is also suggested by controlled studies in several animal species where males contribute nothing more than sperm to offspring. These studies have shown that early life conditions (e.g. nutrition level, toxin exposure and stress) can affect subsequent generations via the paternal lineage [7,10], indicating that the early life environment of males may lead to epigenetic alterations in sperm or male germ cells which are then transmitted to offspring.

It should be noted that ‘true’ epigenetic inheritance has proved challenging to demonstrate when environmental effects operate during pregnancy in mammals, since the definition requires mothers to transmit an environmental signal to offspring, who did not experience the initial stimulus themselves [51]. In such situations, environmental factors affect not only the mother (F_0_) and her fertilized embryos (F_1_), but potentially also the germ cells (embryonic precursors of gametes) that are developing *within* those embryos (figure 1). Thus, the environment is acting directly on precursors of second generation (F_2_) offspring. Accordingly, only epigenetic marks/phenotypes transmitted to F_3_ progeny are said to be inherited inter-generationally, as the developing germ cells that give rise to the F_2_ generation are already present (and thus exposed) during the embryonic development of the F_1_ generation [51]. The majority of mammalian studies that have sought ‘true’ epigenetic inheritance of environmental effects via *in utero* exposure have not found them or produced conflicting results, suggesting that in many cases, epigenetic alterations may only be temporary and that effects on F_2_ offspring can be attributed to germline exposure [7]. However, longer term effects have been reported following toxicological exposure of the fetus [52]. In contrast to these examples, if environmental effects act even earlier in life, i.e. on unfertilized gametes of F_0_ parents, when the germline is not yet established, then true epigenetic inheritance requires only observation of epigenetic/phenotypic changes in F_2_ offspring. This type of transmission has recently been demonstrated in an elegant study on the cross-generational response to olfactory cues in mice [35].

In some cases, trans-generational epigenetic modifications that stem from early life events can be ‘self-perpetuating’ and be repeated across consecutive generations. Cross-fostering experiments in rodents have shown that the type of maternal care behaviour received by a pup during the nursing period will determine the care behaviour devoted by that pup to its own future offspring [53], and comparable patterns of ‘behavioural programming’ that stem from early life behavioural interactions have been reported in humans and avian systems [54,55]. In rodents, this cycle is correlated with epigenetic regulation of glucocorticoid receptors in brain, and similar epigenetic changes have been reported in adult humans who experienced abuse in childhood, suggesting a link between the cyclic transmission of early life events and epigenetic regulation of genes involved in the stress response [49,56].

Despite the likely contribution of epigenetic modifications to the transmission of early life environmental effects from one generation to the next, it would be remiss to ignore the role of non-genomic factors. In egg-laying species, it has been shown that parental exposure to stressors in early life (even prior to hatching) can affect the behaviour of their own offspring [32,33]. While germline epigenetic alterations could be the causal mechanism in these studies, the effects on offspring could also have been brought about by endocrinological changes to the mother that influenced levels of hormones in her eggs, affecting offspring developmental pathways. Early life conditions can also cause long-term structural changes in the maternal phenotype that affect the size and growth trajectories of her offspring (figure 1). In humans, for example, prenatal growth restriction can result in reduced ovarian and uterine size [57], which probably induces an inter-generational cycle of growth effects: girls who experience poor nutrition *in utero* or during early childhood grow to be smaller mothers and in turn give birth to small babies [58–61]. There is evidence for similar effects of juvenile growth trajectories on the size of eggs laid by domesticated and wild species of birds [62–64], and offspring size effects that stem from early life environmental manipulations of parents have been reported in a wide range of organisms from natural populations (table 1). Early life conditions could also influence future generations in other more subtle ways than by direct epigenetic alterations to gametes, the germline or parental physiology. It has recently been proposed that the expression of male secondary sexual characters, such as song and coloration, may reveal the capacity of an individual to cope with developmental stressors and thus allow females to assess the genetic ‘quality’ of potential mates [65]. If females alter their investment in response to such cues [66], early life conditions that induce permanent changes in the expression of male sexual traits (e.g. dietary effects on male plumage [67]) could also have repercussions for the performance of their future offspring ([10,66], figure 1).

## 5. ‘Predicting’ the future from early beginnings?

The concept that the environment is able to ‘instruct’ the parental phenotype in preparing its young, whether adaptively or maladaptively (depending on whose fitness is in question), is intuitively appealing. However, for such trans-generational plasticity to evolve, the benefits of programming offspring phenotypes in this way must outweigh any potential costs. One such cost is the advance commitment to a particular phenotype, since environmentally induced phenotypic changes are often irreversible. Thus, the accuracy of environmental cues in predicting coming environments, either within a generation or across them, is paramount for the evolution of plasticity [8,68]. Such issues have been addressed in several recent theoretical models, which can be broadly categorized as being based on either ‘external’ or ‘internal’ modes of environmental prediction. External prediction occurs when offspring phenotypes are programmed according to an exogenous cue, e.g. photoperiod or temperature, which is perceived by the parents. This type of model was first conceptualized as the classic ‘maternal effect’ described by Mousseau & Fox [9] and more recently it has been expanded within the context of epigenetic inheritance as a ‘detection-based effect’ [69]. Similar concepts, such as the ‘external predictive adaptive response’ [70] or ‘environmental morph determination’ [71], have been proposed for within-generation phenotypic plasticity, but are readily extendable to account for cross-generational phenotypic effects.

In these external prediction models, individuals are required to make developmental decisions about future conditions (e.g. for their young) that are based entirely upon cues derived from the external environment early in their life. Intuitively, this seems more plausible in short-lived organisms, where the probability of the cue experienced in early life being a valid predictor of the offspring environment should be higher. Empirical evidence for this mode of external prediction within the context of early life effects comes from a study where larval fruit flies were raised on poor- or high-quality food as larvae, and then switched to a standard quality diet before they matured and laid eggs. F_1_ offspring were then reared on poor- or high-quality food themselves. Offspring raised on poor food pupated earlier if their parents had also been raised on poor food, whereas if the offspring were reared on good food, then parental rearing diet had no effect on pupation time [24]. In this example, the accuracy of the cue experienced by the parents as larvae is likely to be high because food availability may vary little over a timescale of days.

However, in longer lived species, or for exposures to unfertilized gametes, the relevance of external prediction is less clear, with debate regarding its role in human life-history evolution being particularly polarized (e.g. [72,73)]. For instance, it has been suggested that the correlation between early life and adult environments in humans would have to be nearly perfect to favour the evolution of adaptive plasticity in reproductive timing and that this correlation is likely to be even more restrictive for inter-generational effects [70]. Indeed, if this were the case, plastic strategies would actually become redundant, particularly if they are associated with any costs [70].

Given the apparent shortcomings associated with external modes of environmental prediction as a general explanation for the evolution of adaptive plasticity that stems from early life conditions, Nettle *et al*. [70] proposed that programming decisions should have evolved to use as broad a sampling window and as diverse a range of cues as possible. Internal modes of prediction represent one such possibility: these differ in that ‘cues’ embodied within an individual's genotype, epigenotype or somatic state are used to instruct developmental decisions. For example, owing to a history of selection an individual's genotype should contain information about the recent local environment, which could serve as a predictor of a given phenotype's likely success in the near future and thus act as an internal input to the developmental process [71]. A similar concept has been proposed for epigenetic states that have a history of stable transmission across generations [69].

Possible evidence for such methods of internal prediction comes from species with complex life cycles, where juvenile and adult ecologies can differ greatly due to ontogenetic niche shifts, dispersal, migration or prolonged offspring development [74]. Accordingly, parents may be unable to reliably predict offspring conditions from environmental cues at the time of mating (especially if gestation or incubation is prolonged). However, their own experiences as juveniles may allow them to predict their offspring's future environment [27,75]. For example, in the cichlid fish, *Simochromis pleurospilus*, juveniles inhabit shallower more productive water, using only a narrow range of depths, whereas mature females use deeper habitats. In an experiment that performed factorial cross-overs between the juvenile and adult environments of the parents, Taborsky [27] demonstrated that mothers who were subjected to food restriction as juveniles subsequently produced larger, faster growing offspring, irrespective of their access to food after sexual maturity. Owing to the strong positive relationship between offspring size and performance in adverse environments [76,77], it was inferred that female cichlids growing up under conditions of low food were ‘preparing’ their offspring for a similarly poor environment themselves.

Related to these variants of internal prediction is the concept of the internal predictive adaptive response (internal PAR, [70]), which was developed to describe the acceleration of reproductive timing that occurs in humans subjected to early life adversity. In this model, the early life environment shapes the somatic ‘state’ of the individual through to adulthood, which in turn affects its optimal pattern of reproductive investment (e.g. if an adverse early environment reduces adult life expectancy, then the optimal age of sexual maturity is decreased). An advantage of the internal PAR concept is that it is not dependent on a reliable correlation of early life environments from one generation to the next but instead on the more realistic scenario that environmental conditions in early life affect the physiological state of the adult [70]. Although this model was developed to describe within-generation plasticity in response to early life stress, it can be extended to inter-generational effects [70] (as any change in reproductive investment is also likely to affect the phenotype of the offspring) and potentially to other types of environmental stimuli—both positive and negative. Thus, the advantage of the internal PAR is that it can account for ‘best of a bad job’ scenarios where parents might favour their own fitness at the expense of offspring and also it does not exclude the input of external predictors, nor of the other internal predictors. It is presumed that internal PAR modes of prediction are likely to be more prevalent in longer lived species, where the accuracy of external cues in predicting offspring conditions years or decades later is likely to be low. Possible examples of internal PARs include the inter-generational transmission of metabolically impaired phenotypes to grand-offspring following fetal adversity in rodents (see references in [7]), whereby the development of poor somatic state by the mother might result in her sacrificing the individual ‘quality’ of her offspring to increase her own chances of survival and hence lifetime reproductive success.

## 6. Designing and analysing future studies

We are only beginning to understand the generation-spanning effects of early life experiences, but it is clear that they can be diverse and long-lasting, and have clear ecological relevance since in many species reproduction is synchronized and so adverse environmental conditions at key moments in development could affect entire cohorts. Presently though, the ecological implications of inter-generational effects of early life conditions remain unclear due to both conceptual and methodological issues. Several of these issues, chiefly the infrequent use of fully factorial experimental designs (i.e. designs that manipulate both parent *and* offspring environments), a tendency to focus only on offspring fitness outcomes and the prevalence of ‘snap-shot’ measurements of offspring, have been addressed in previous treatments of the topic [78,79]. However, we feel that there is an additional methodological problem that is specific to the type of parental effects addressed here and one that could be easily rectified: trans-generational effects of early life conditions tend to be presumed if a phenotypic response is observed in offspring whose parents (or grandparents) were subjected to an experimental manipulation during their own development and then transferred to control conditions before reaching sexual maturity. This presumption may be erroneous, however, because any response in offspring might be induced by the existence of a contrast between natal and adult environments of their parents. Ideally, parents should be assigned alternately to treatment or control groups in early life. Then, upon reaching sexual maturity, treatment and control parents should be either switched to contrasting conditions or maintained in an environment resembling the conditions they experienced in early life. In order to determine the fitness consequences, their offspring should then also be raised in the two contrasting environments. Although more logistically demanding, such ‘cross-over’ manipulations between the natal and adult environments of the parental generation have revealed that early life conditions experienced by parents can influence offspring development irrespective of the environment experienced in adulthood [24,27,34,80,81]. This type of experimental approach can identify any effect of the early life of the parent on offspring, but crucially, can also reveal any confounding effects on offspring that might arise from switching between different juvenile and adult environments of the parent (e.g. due to catch-up or compensatory growth).

Here, we have outlined a conceptual framework for understanding the ecological context of cross-generational effects that stem from early life experiences. Principally, we focused on the importance of environmental predictability/cue accuracy to illustrate how parents might use a broad range of cues when investing in young. However, this framework does not formally address the potential costs of such plastic responses to parents/offspring, the estimations of which vary (e.g. [70,82]) and the implications of which are potentially large. Further theoretical advancement could incorporate several other factors that are likely to modulate the end-product of such early life effects: parents and their young will not necessarily ‘agree’ over the optimal offspring phenotype to result from environmentally induced early life effects and thus offspring may also respond via counter-strategies of their own [79].

Despite widespread consensus regarding the importance of environmental predictability for adaptive plastic responses to evolve, to our knowledge, controlled experimental tests of this hypothesis have not been performed. Given the particular relevance of this issue to the current topic and the evolution of transgenerational plasticity in general [68], there is no reason why the generation-to-generation correlation of early life environments cannot be manipulated empirically and treated as a covariate when analysing the cross-generational outcomes of early life effects. In terms of mechanisms, our understanding of epigenetic inheritance processes is largely specific to mammals and plants, meaning that their relevance in perpetuating early life effects across generations in other organisms is unclear at present, an issue compounded by the scarcity of experimental data that extend beyond the F_2_ generation in non-rodent and plant systems (table 1). However, with increased understanding of how epigenetic processes mediate the inter-generational effects of early life conditions, we may be better placed to make epigenetic manipulations of the parental phenotype (e.g. via methylation inhibitors such as 5-azacytidine, [83]) that might offer a starting point to begin disentangling the relative roles of external and internal modes of prediction in facilitating the inter-generational effects of early life experience. The inter-generational consequences of early life effects are of immense interest to researchers from many different biological sub-disciplines ranging from the ecologist who might wish to understand the long-term repercussions of natal habitat variation on population dynamics, to the epidemiologist aiming to stem the transmission of cardio-vascular or metabolic diseases from parent to child or grandchild via targeted intervention programmes. We hope that our article will stimulate further studies in this area, so that the broad-scale implications of these phenomena will be better understood.
